# A large dataset of synthetic SEM images of powder materials and their ground truth 3D structures

**DOI:** 10.1016/j.dib.2016.10.011

**Published:** 2016-10-22

**Authors:** Brian L. DeCost, Elizabeth A. Holm

**Affiliations:** Department of Materials Science and Engineering, Carnegie Mellon University, USA

**Keywords:** Computer vision, Computational materials science, Image databases, Metal powder, Microstructural analysis

## Abstract

This data article presents a data set comprised of 2048 synthetic scanning electron microscope (SEM) images of powder materials and descriptions of the corresponding 3D structures that they represent. These images were created using open source rendering software, and the generating scripts are included with the data set. Eight particle size distributions are represented with 256 independent images from each. The particle size distributions are relatively similar to each other, so that the dataset offers a useful benchmark to assess the fidelity of image analysis techniques. The characteristics of the PSDs and the resulting images are described and analyzed in more detail in the research article “Characterizing powder materials using keypoint-based computer vision methods” (B.L. DeCost, E.A. Holm, 2016) [1]. These data are freely available in a Mendeley Data archive “A large dataset of synthetic SEM images of powder materials and their ground truth 3D structures” (B.L. DeCost, E.A. Holm, 2016) located at http://dx.doi.org/10.17632/tj4syyj9mr.1[2] for any academic, educational, or research purposes.

**Specifications Table**

**Table:** 

Subject area	*Materials Science, Computer Science*
More specific subject area	*Microstructural image analysis and classification*
Type of data	*Images, 3D structures, scripts*
How data was acquired	*Simulated 2D SEM images were rendered from the ground truth 3D structures using the Blender*[3]*open source graphics rendering suite*
Data format	*Raw 3D structures in.JSON plain text format, processed digital images in.PNG format, formatted scripts in.SH and.PY plain text formats*
Experimental factors	*Spherical particles are placed at random with size drawn at random from the generating particle size distribution. The resulting structures are rendered at a resolution of 512 × 512 pixels.*
Experimental features	*2048 images of powder materials, representing 8 related but distinct particle size distributions*
Data source location	*Pittsburgh, PA, USA*
Data accessibility	*Data are publicly available via Mendeley Data at*http://dx.doi.org/10.17632/tj4syyj9mr.1

**Value of the data**

• **Microstructural image analysis is a core discipline and an active research area in materials science; however, data science approaches to microstructural image analysis are hindered by a lack of large, well-understood image data sets. The images in this data set help fill that gap by providing a statistically significant number of powder material images with known ground truth characteristics.**
• **Because their generating particle size distributions are closely related, the resulting structures are challenging to differentiate, thus they present a useful benchmark to assess the fidelity of image analysis techniques.**
• **When combined with their ground truth structures and classifications, these images can be used to benchmark image analysis approaches including segmentation, quantitative characterization, machine learning, and others.**
• **Images of powder materials are especially important for understanding powder bed based Additive Manufacturing (AM) processes.**

## 1 Data

**Fig. 1:**
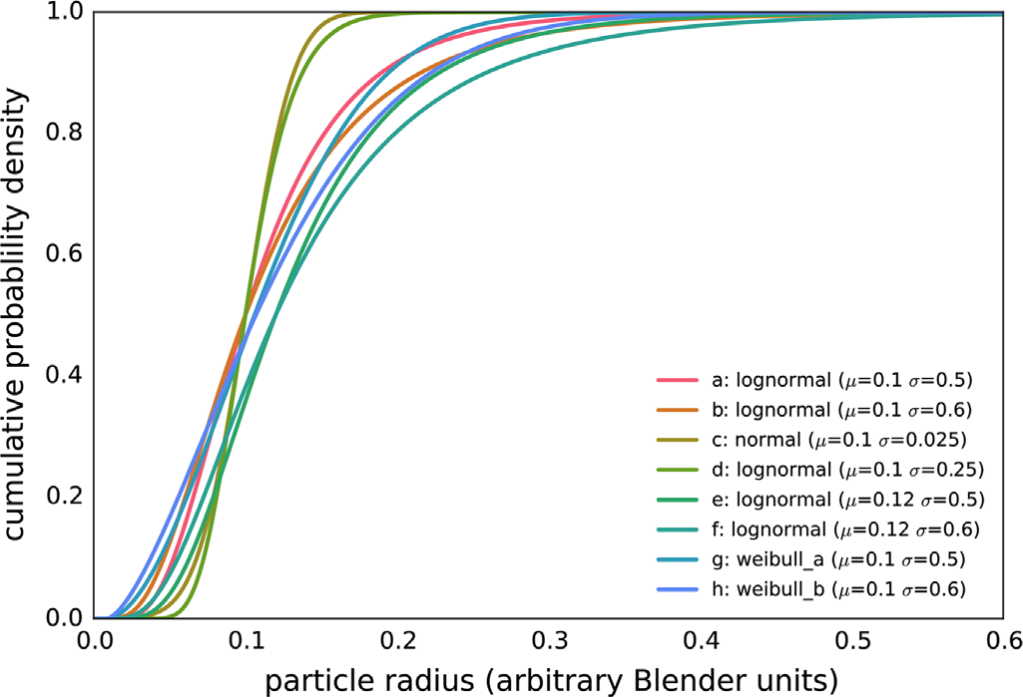
Cumulative probability distributions for the eight particle size distributions sampled to create the synthetic SEM images in this dataset.

**Fig. 2:**
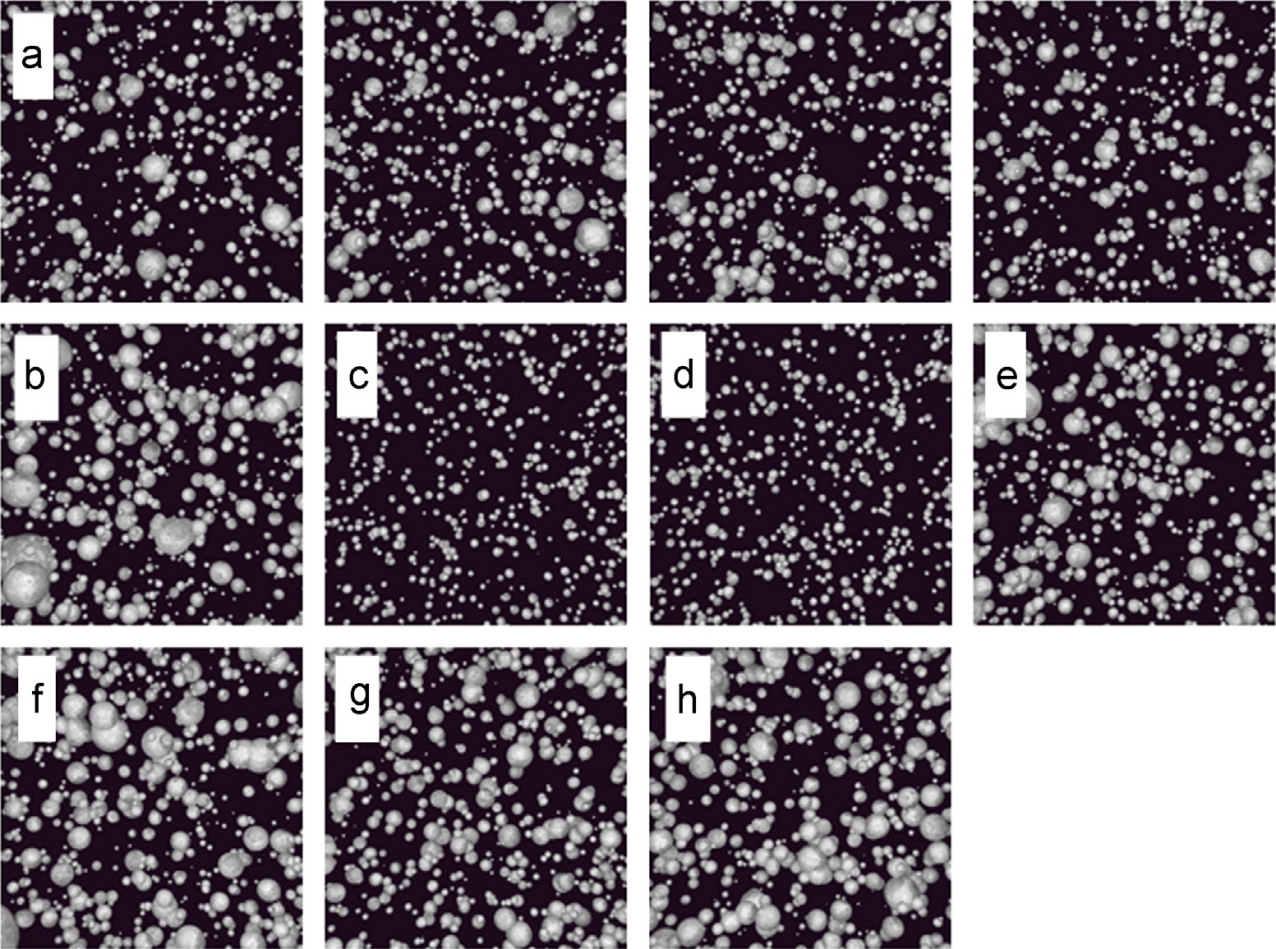
Example synthetic SEM images of powder materials. (a) Four examples representing particle size distribution ‘a,’ as shown in Fig. 1. (b–h) One example each of particle size distributions ‘b’ through ‘h’.

**Fig. 3:**
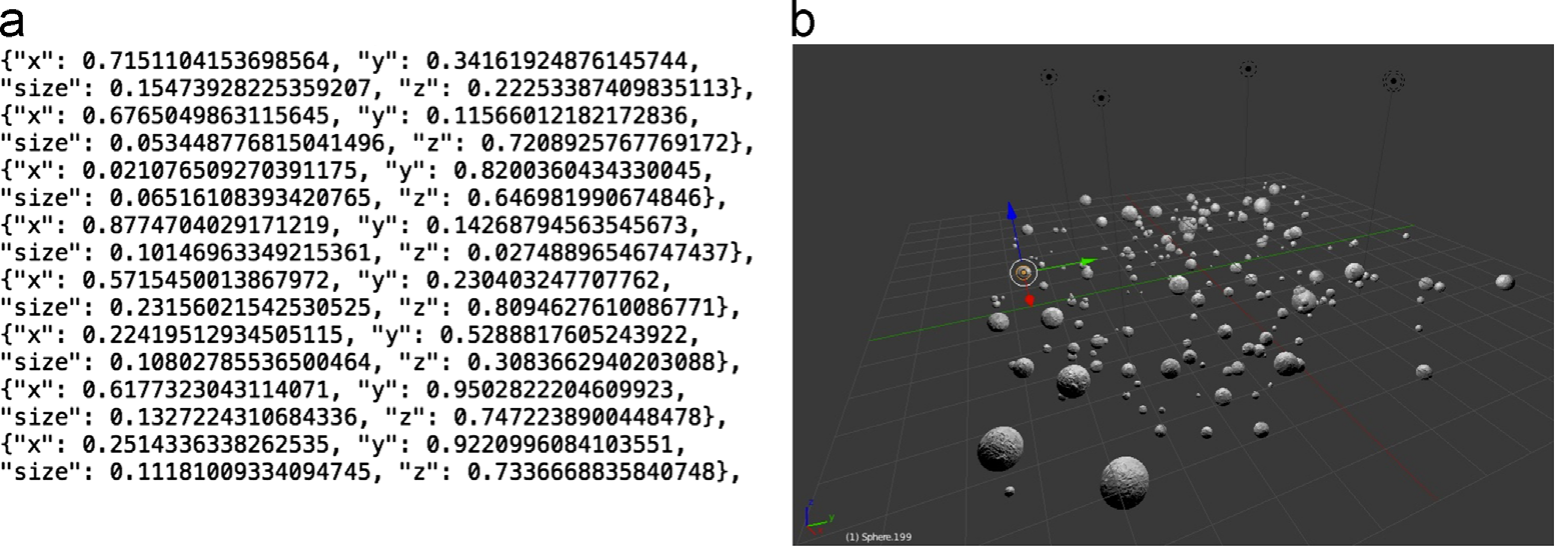
Example of a 3D ground truth particle structure. (a) A text snippet from the.JSON structure file, showing how particle sizes (“size”) and coordinates (“x”, “y”, and “z”) are represented. (b) A 3D rendering of a particle structure, which would be used to create a synthetic 2D SEM image as described in the text. The x-axis is shown by the red arrow; the y-axis is the green arrow; and the z-axis is the blue arrow. The imaging plane is located at *z*=0, and is shown by the overlaid grid.

This data set is comprised of 2048 synthetic scanning electron microscope (SEM) images of powder materials and descriptions of the corresponding 3D structures that they represent [2]. There are 256 images/structures from each of eight closely related particle size distributions (PSDs), as described in Table 1. Fig. 1 shows the PSDs sampled to create the images, and Fig. 2 shows example images from each PSD. Fig. 3 shows a text snippet from a 3D structure file, as well as a rendering of an example 3D powder structure that would be synthetically imaged to generate a 2D micrograph.

## 2 Experimental design, materials and methods

The dataset of 3D structures and their corresponding 2D images was created using Blender [3], an open source computer graphics suite used for 3D modeling, rendering, animation, and scientific visualization. In this dataset, powders are comprised of spherical particles with sizes drawn at random from the appropriate PSD. We consider eight PSDs, as shown in Fig. 1, and construct 256 independent structure/image pairs for each PSD, resulting in 2048 synthetic powder micrographs (examples are shown in Fig. 2).

To synthesize each image, we use an 11×11×2 (arbitrary Blender units) render volume and insert 800 particles placed at random. Particle radii are selected at random from one of the eight generating PSDs, and they are permitted to intersect and/or occlude each other. Particles are rendered using a spherical mesh, with a surface texture achieved by wrapping the particle with an image of zinc grains, included in the dataset. The particles are imaged on the *z*=0 plane, which intersects the centroid of the render volume, as shown in Fig. 3(b). The camera is located in the center of the volume at height *z*=10, and the resulting image resolution is 512 × 512 pixels. Python scripts used to perform these operations are included in the dataset files.

It is worth noting that the PSDs in Fig. 1 consist of four pairs of size distributions that are relatively similar to each other. The characteristics of the PSDs are given in Table 1 and described in more detail in [1].

## Figures and Tables

**Table 1:** Dataset file structure.
