# Corrigendum to “A large dataset of synthetic SEM images of powder materials and their ground truth 3D structures” [Data Brief 9 (2016) 727–731]

**DOI:** 10.1016/j.dib.2017.07.020

**Published:** 2017-07-13

**Authors:** Brian L. DeCost, Elizabeth A. Holm

**Affiliations:** Department of Materials Science and Engineering, Carnegie Mellon University, USA

The authors regret that an incorrect grant number appeared in the Acknowledgements. The correct funding statement should read as follows:

We gratefully acknowledge funding for this work through National Science Foundation grants DMR-1307138 and DMR-1507830, and through the John and Claire Bertucci Foundation.

The authors would like to apologise for any inconvenience caused.

